# Cardiac output measurements via echocardiography versus thermodilution: A systematic review and meta-analysis

**DOI:** 10.1371/journal.pone.0222105

**Published:** 2019-10-03

**Authors:** Yun Zhang, Yan Wang, Jing Shi, Zhiqiang Hua, Jinyu Xu

**Affiliations:** 1 Department of Emergency Medicine, Wuxi People’s Hospital Affiliated to Nanjing Medical University, Wuxi, Jiangsu, China; 2 Education Department, Wuxi People’s Hospital Affiliated to Nanjing Medical University, Wuxi, Jiangsu, China; Scuola Superiore Sant'Anna, ITALY

## Abstract

Echocardiography, as a noninvasive hemodynamic evaluation technique, is frequently used in critically ill patients. Different opinions exist regarding whether it can be interchanged with traditional invasive means, such as the pulmonary artery catheter thermodilution (TD) technique. This systematic review aimed to analyze the consistency and interchangeability of cardiac output measurements by ultrasound (US) and TD. Five electronic databases were searched for studies including clinical trials conducted up to June 2019 in which patients’ cardiac output was measured by ultrasound techniques (echocardiography) and TD. The methodological quality of the included studies was evaluated by two independent reviewers who used the Quality Assessment of Diagnostic Accuracy Studies-2 (QUADAS-2), which was tailored according to our systematic review in Review Manager 5.3. A total of 68 studies with 1996 patients were identified as eligible. Meta-analysis and subgroup analysis were used to compare the cardiac output (CO) measured using the different types of echocardiography and different sites of Doppler use with TD. No significant differences were found between US and TD (random effects model: mean difference [MD], -0.14; 95% confidence interval, -0.30 to 0.02; *P* = 0.08). No significant differences were observed in the subgroup analyses using different types of echocardiography and different sites except for ascending aorta (AA) (random effects model: mean difference [MD], -0.37; 95% confidence interval, -0.74 to -0.01; *P* = 0.05) of Doppler use. The median of bias and limits of agreement were -0.12 and ±0.94 L/min, respectively; the median of correlation coefficient was 0.827 (range, 0.140–0.998). Although the difference in CO between echocardiography by different types or sites and TD was not entirely consistent, the overall effect of meta-analysis showed that no significant differences were observed between US and TD. The techniques may be interchangeable under certain conditions.

## Introduction

Continuous or dynamic cardiac function monitoring plays a crucial role in the diagnosis, assessment, treatment, and prognosis of critically ill patients. Cardiac output (CO) measurement is one of the most important parameters in cardiac function monitoring. The commonly used CO measurement methods include indirect Fick methods, thermodilution (TD), Doppler ultrasound (US) or echocardiography, partial carbon dioxide (CO_2_) rebreathing, thoracic electrical bioimpedance, and magnetic resonance imaging [1, 2]. TD via the pulmonary artery (PA) catheter is still considered to be the gold standard method in the clinical setting. However, this method has disadvantages because it is invasive and can lead to severe complications [3]. Echocardiography, as a noninvasive or semi-invasive method for the assessment of cardiac anatomy and function, is favored in clinical practice. The methods commonly used for echocardiography include transthoracic echocardiography (TTE), transesophageal echocardiography (TEE), ultrasonic CO monitor (USCOM), noninvasive continuous CO system (NICO), and ultrasound dilution (UD). Several sites can be used for CO measuring. The velocity time integral (VTI) and cross-sectional area (CSA) of the ascending aorta (AA) [4], PA [5], aortic valve (AOV) [6], mitral valve [7], or left ventricular outflow tract (LVOT) [8]can be used to calculate the stroke volume (SV) using CSA×VTI and the CO = SV×heart rate. Simpson’s rule [9] was the first method to delineate the innermost endocardial border of the left ventricle using the trackball at end systolic and end diastolic and then to calculate the left ventricular end-diastolic volumes (LVEDV) and left ventricular end-systolic volumes (LVESV): CO = (LVEDV-LVESV)×heart rate. Some studies used the common carotid artery in point-of-care US to estimate the CO [10]. UD [11] technology is also used to measure hemodynamic variables based on the Stewart-Hamilton principle. This method utilizes an extracorporeal arteriovenous tubing loop (AV loop) inserted between existing arterial and venous catheters and isotonic saline as an indicator [12].

Whether echocardiography can replace TD method in CO measurement remains controversial. Some studies [13–15] have revealed that echocardiography is a rapid, accurate, and noninvasive monitoring technology suitable for patients in ICU. Although differences were observed, some studies [10, 16] showed the correlation was good. However, some studies [17, 18] suggested that echocardiography is not interchangeable with TD for measuring CO. Therefore, this study aimed to evaluate the consistency and interchangeability of cardiac output measurements in US and TD and to find the most optimal types or sites used of echocardiography for CO measuring if possible.

## Materials and methods

This review was performed according to the Preferred Reporting Items for Systematic Reviews and Meta-Analysis of diagnostic test accuracy (PRISMA-DTA) statement. Ethics committee approval was not required, as it was a review of published data.

### Search strategy

An electronic literature search was performed in PubMed, EMBASE (using OVID), Cochrane Controlled Trials Registry, China National Knowledge infrastructure, and Wanfang Data from their inception up to June 2019. The EndNote X6 software (Thomson Reuters Corporation, New York, NY, USA) was used to eliminate duplicates and manage these citations. The following search strategy was used to identify studies:

1. transtho*[Title/Abstract] OR transeso*[Title/Abstract] OR echocard*[Title/Abstract] OR cardiac ultrasound [Title/Abstract] OR Doppler [Title/Abstract] OR USCOM [Title/Abstract]
2. cardiac output [Title/Abstract]) AND thermodilution [Title/Abstract]
3. #1 AND #2

### Study selection

The inclusion criteria were (1) critically ill patients, (2) clinical trials, (3) studies that used echocardiography to measure the CO, (4) studies that used TD technique as the reference technique, and (5) studies in which outcomes of interest included the data of CO or all the differences between the techniques (bias) and standard deviations (SDs) or bias and limits of agreement (LOA).

The exclusion criteria were (1) reviews or case reports, (2) animal studies, (3) studies published in languages other than English, (4) studies only published as an abstract, and (5) studies with no mean and SD of CO and without bias and LOA/SD between two techniques.

Study selection and data extraction were performed by two independent reviewers (YZ and JS). Disagreements were resolved through consensus.

### Data extraction

A data collection form was developed prior to data extraction. Two authors (YZ and YW) extracted relevant data from included articles. The extracted data included (1) first author and year of publication; (2) number of patients, sex, and age; (3) the data of CO in both groups; (4) the type of ultrasound and sites; (5) the bias, SD, LOA, and percentage error (PE); (6) the Pearson R coefficient and linear equations; and (7) patient population.

When the results of the trial were reported as median and quartile, the Stela Pudar-Hozo method was used to estimate the mean and standard deviation. Bias was defined as the mean of the two measurement differences, and LOA was defined as bias±1.96SD (some studies defined LOA as bias±2SD). The PE was defined as 1.96SD divided by the mean CO of the two methods. Posteriori probability was also calculated.

Ethics approval was waived for this study as patient consent was obtained within the individual trials and all data were anonymized.

### Quality assessment

Studies with critically ill patients who needed CO monitoring were included. The CO measured by thermodilution was the reference standard, regardless of other modes of CO monitoring. Study quality was assessed using QUADAS-2, which was tailored for our systematic review (S1 Table). The quality of each paper was evaluated by two authors (YW and JS) independently, and any disagreements were resolved by consensus.

### Statistical methods

The systematic reviews were conducted in compliance with the PRISMA guidelines. The Review Manager Software version 5.3 for Windows (The Cochrane Collaboration, 2014) was used to perform the meta-analysis. The STATA version 12.0 (StataCorp, College Station, TX, USA) was used to analyze the publication bias (Egger’s test). The Cochrane Q-test was used for heterogeneity analysis. A fixed-effect model was used when *P*>0.1 and I^2^<50%; otherwise, a random effects model was adopted. If necessary, a sensitivity analysis was performed to reduce the heterogeneity to *P*>0.1 and I^2^<25% by omission of some studies as few as possible. All P-values were two-sided, and P<0.05 was considered significant.

## Results

### Search results

**Fig 1:**
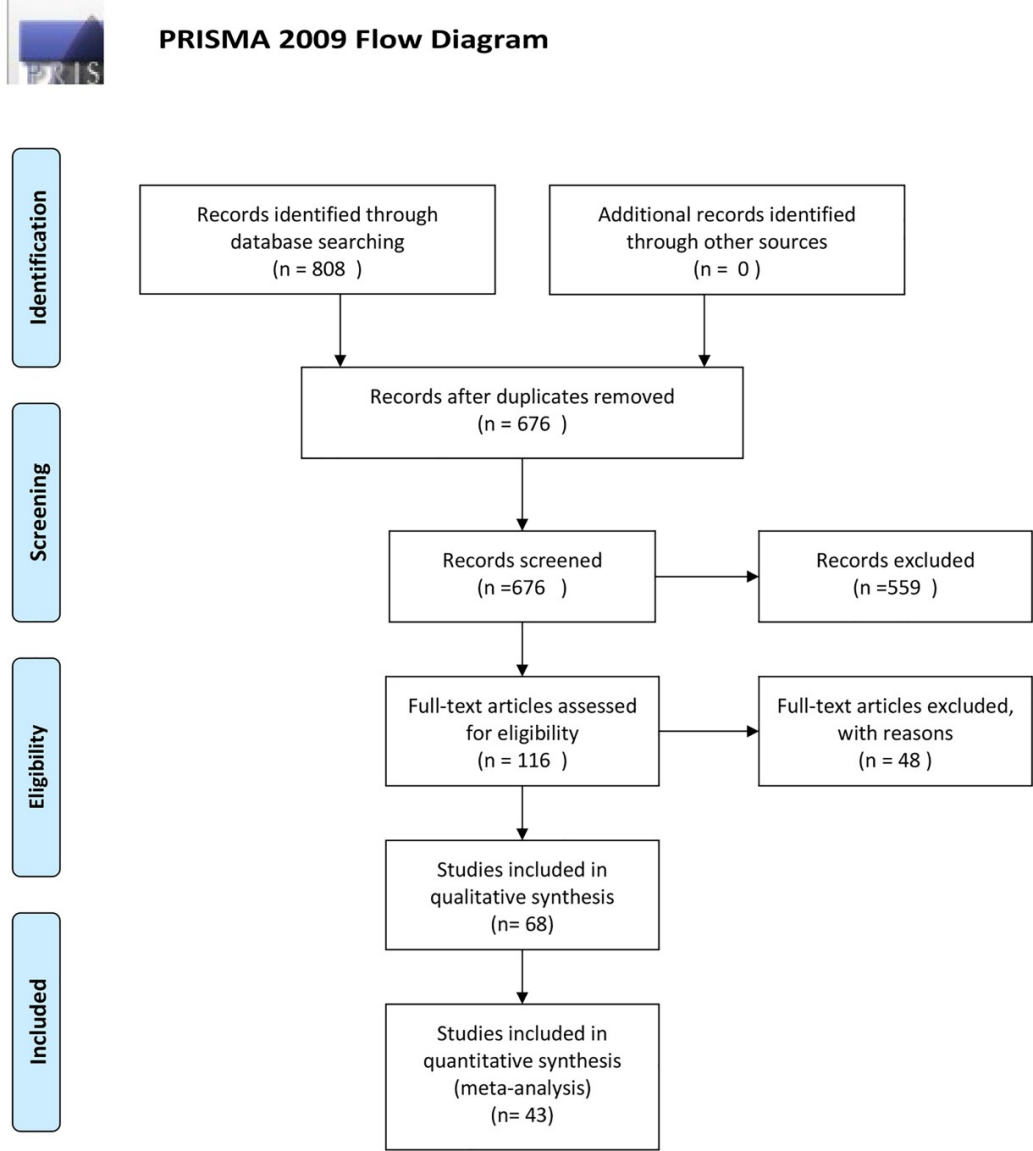
Flowchart of identification of eligible studies.

Of the initial 808 records identified, 676 remained after duplicates were removed. Then, 477 records were excluded based on the title, and 83 records excluded based on the abstract; 116 articles were evaluated in full text. Forty-eight full-length articles were excluded, including 33 articles with missing data and 15 articles that used TD and US techniques, but not CO measurement, in the optimization of cardiac preload. Finally, 68 and 43 studies were included in qualitative synthesis and quantitative synthesis, respectively (meta-analysis) (Fig 1). All studies were published between 1971 and 2018 (Table 1).

**Table 1:** Main characteristics of the included studies.

Studies	N	Types of US	Sites	LOA		Bias	PE(%)	R	Linear equation or Notes	Population
Arora 2007[19]	30	USCOM	AA	-0.86	0.59	0.13				OPCAB
Axler 1996[20]	13	TEE	Simpson	-4.00	4.60	-0.30	26.0	0.680		Mechanically ventilated critically ill patients
Basdogan 2000[21]	33	ACM	LVOT	-2.35	2.52	-2.35		0.570	COUS = 0.35COTD+3.55	Intensive care patients
Beltramo 2016[22]	31	USCOM	AoV	-1.20	1.60	0.20	11.0	0.870		Pediatric patients for heart transplantation, dilated/ hypertrophic / restrictive cardiomyopathy
Bojanowski 1987[23]	12	TTE	AoV	0.10	1.20	0.60		0.880	COUS = 1.26+0.87COTD	CHF, PH, MVD
Botero 2004[24]	68	NICO	AA	-2.10	2.20	0.04	44.8			CABG
Cariou 1998[25]	20	USCOM	DA			-2.31		0.800		Critically ill patients with mechanical ventilation
Castor 1994[2]	10	TTE	AA			-0.70	16.0		IPPV	ASA-PS Ⅲ-Ⅳ
Castor(1) 1994[2]	10	TTE	AA			-0.70	18.7		Apnoea	ASA-PS Ⅲ-Ⅳ
Castor(2) 1994[2]	10	TTE	AA			-2.50	32.4		Spontaneous ventilation	ASA-PS Ⅲ-Ⅳ
Chand 2006[4]	50	USCOM	AA	-1.69	1.41	-0.14				OPCAB
Chandraratna 2002[26]	50	TTE	PA	-0.48	0.96	0.24		0.920	COUS = 0.93COTD+0.60	Patients in the coronary care department for treatment of CHF or hemodynamic instability
Chew 2009[6]	12	TEE	AoV			0.06				Severe sepsis and septic shock in the medical ICU
Coats 1992[27]	6	TTE	AA	-1.71		-0.40			TD>DU	Ischemic heart disease, CHF or PH
Corley 2009[28]	30	USCOM	AA	-1.40	0.70	0.34		0.848		Evaluation for CHF and/or PH
Crittendon 2012[29]	28	UD	AV loop	-0.81	0.80	-0.01	25.4	0.950	COUS = 0.92COTD+0.26	Cardiac transplantation, PH
Darmon 1994[30]	63	TEE	AoV	-0.77	0.89	-0.06	19.0	0.940	COUS = 0.94COTD+0.19	CABG or automatic cardioverter defibrillator insertion
Descorps-Declere 1996[31]	28	TEE	LVOT	-1.73	0.89	-0.42	16.0	0.975	COUS = 0.889COTD+0.74	Acutely ill patients with Swan-Ganz catheter, controlled ventilation, sedation and a stable hemodynamic condition
Dicorte 2000[32]	34	TEE	AA	-0.18	1.16	0.49		0.748	COUS = 1.144COTD-1.625*	CABG
Eremenko 2010[33]	26	UD	AV loop	-2.63	2.62	0.00	22.2	0.910	COUS = 0.93COTD+0.42	Adult post cardiac surgery patients
Estagnasie 1997[7]	22	TEE	MV	-3.40	2.80	0.30		0.780	COUS = 0.93COTD+0.76	Mechanically ventilated patients
Feinberg 1995[34]	29	TEE	LVOT	-1.10	1.30	-0.10	25.0	0.910	COUS = 0.97COTD-0.03*	Undergone open heart surgery, acute myocardial infarction
Froese 1991[35]	7	TTD	AA	-6.40	12.48	3.04		0.140		Patients for elective surgery under general anaesthesia
Galstyan 2010[36]	30	UD	AV loop	-1.72	1.65	0.04	20.0	0.950	COUS = 1.03COTD-0.24	Hematology ICU
Gassner 2015[10]	36	POCUS	CCA	-2.12	2.58	-0.23		0.815		surgical and cardiothoracic ICU
Hammoudi 2016[8]	15	3D-TEE	LVOT	-2.37	3.33	0.48	53.0	0.720		ICU patients on mechanical ventilation
Hammoudi(1) 2016[8]	15	TEE	LVOT	-1.97	2.74	0.38	44.0	0.780		ICU patients on mechanical ventilation
Hausen 1992[37]	9	TTD	AA	-1.56	4.99	1.70		0.248	COUS = 0.126COTD+0.81	Patients after open heart surgery
Hoole 2008[38]	36	RT-3DE	Simpson	-0.84	0.72	-0.06		0.910	COUS = 0.86COTD+0.45	Cardiac transplant assessment
Horster 2012[39]	70	USCOM	TPF/TAF	-2.34	1.62	0.05	29.0	0.890		Septic patients
Horster-1 2012[40]	20	USCOM	TPF/TAF	-2.94	3.98	0.52	13.0			Mechanically ventilated (PEEP≤10mbar) adult patients with pneumonia and septic shock
Huntsman1983[41]	45	TTE	AA	-1.02	1.26	0.12	17.0	0.940	COUS = 0.95COTD+0.38	ICU patients
Izzat 1994[42]	21	TEE	LVCSA	-3.87	4.81	0.47		0.450		Patients undergoing open heart operations
Izzat(1) 1994[42]	21	TEE	PA	-0.78	1.02	0.12		0.950		Patients undergoing open heart operations
Knirsch 2008[43]	24	USCOM	AoV	-1.47	1.21	-0.13	36.4			Pediatric patients with CHF
Lee 1988[44]	16	TTE	AoV	-0.28	0.15	-0.07		0.940	COUS = 1.35COTD-1.91*	Sever pregnancy-induced hypertension, eclampsia, hemorrhagic shock, renal failure
Levy 1985[45]	26	TTE	AA	-0.11	0.91	0.40		0.960	COUS = 0.86COTD+0.29	ICU patients including sepsis, pancreatitis, severe pneumonia and cardiac failure
Marcelino 2006[46]	41	TTE	AoV	-1.80	0.60	-0.58	16.0	0.970	COUS = 0.859COTD+0.47	Post liver transplant patients
Mark 1986[47]	16	TEE	AA					0.919	COUS = 1.05COTD+0.000	Undergoing cardiac surgery
Maslow 1996[48]	38	TEE	AoV	-0.45	0.45	0.01		0.970	COUS = 1.03COTD-0.12	Adult cardiac surgery patients undergoing general anaesthesia
Mayer 1995[49]	48	TTE	LVOT	-2.09	0.59	-0.75		0.670		Aneurysmal clipping
McLean 1997[50]	18	TTE	LVOT	-1.50	1.90	0.20		0.930		Pulmonary embolus, cardiogenic shock, septic shock, Legionnaire’s disease and perioperative myocardial infarction
Missant 2008[51]	20	TTE	AoV	-1.49	2.38	-0.70	43.0	0.730	COUS = 1.58COTD-0.13	OPCAB
Moller-Sorensen 2014[18]	25	TEE	LVOT	-1.73	1.29	0.20	38.6			CABG
Moxon 2003[52]	13	TEE	DA	-2.35	1.89	-0.23		0.810		Cardiac surgery patients
Muhiudeen 1991[53]	35	TEE	PA	-2.70	1.30	-0.70	15.0	0.650	COUS = 0.64COTD+0.97	Patients undergoing cardiovascular surgery
Parra 2008[54]	50	TEE	LVOT	-1.21	1.22	0.04	29.1	0.900		Patients for elective cardiac surgery with CPB
Perrino 1998[55]	32	TEE	AoV	-1.20	1.08	-0.01	24.0	0.910		Patients for either cardiac or noncardiac surgery need for PAC
Pinto 1994[56]	8	TEE	Simpson	-2.80	2.40	-0.20		0.710	COUS = 0.64COTD+1.57	Patients undergoing cardiac surgery
Poelaert 1999[57]	45	TEE	LVOT			-0.54		0.870	TEE pwt	CABG
Poelaert(1) 1999[57]	45	TEE	LVOT			-0.31		0.870	TEE pwl	CABG
Poelaert(2) 1999[57]	45	TEE	LVOT			0.21		0.820	TEE cwt	CABG
Poelaert(3) 1999[57]	45	TEE	LVOT			0.39		0.840	TEE cwl	CABG
Pombo 1971[58]	9	TTE	NR			0.08		0.881	COUS = 0.932COTD+0.48	Myocardial infarction
Ryan 1992[59]	12	TEE	MV	-4.10	2.40	-0.86		0.700	COUS = 0.954COTD+1.14	Undergoing elective major vascular surgery, either aortic aneurysm resection or aorta bifemoral grafting
Sato 2018[60]	12	TEE	PA							Aortic valvular regurgitation, aortic stenosis.
Savino 1991[5]	33	TEE	PA	-0.97	1.02	0.03	24.0	0.930	COUS = 1.096COTD-0.336	Cardiac surgical patients
Segal 1991[61]	20	Dollper PAC	PA	-1.68	1.42	-0.13	25.0	0.760	COUS = 0.87COTD+0.44	Valvular and nonvalvular cardiac surgery, major intraabdominal vascular surgical procedures
Shimamoto 1992[62]	65	TEE	MV	-2.53	0.83	-0.85				After open heart surgery
Shimamoto-1 1992[63]	42	TEE	MV	-0.12	0.06	-0.03		0.930	COUS = 0.90COTD+0.12*	Myocardial infarction, angina pectoris, after CABG
SoutoMoura 2017[64]	15	TTE	LVOT	-0.22	0.28	0.03		0.998		Cardiac arrest with hypothermia
SoutoMoura(1) 2017[64]	15	TTE	LVOT	-1.60	0.75	-0.43		0.843		Cardiac arrest with hypothermia
Su 2008[65]	15	USCOM	AoV	-0.65	0.92	0.13	8.9	0.988	COUS = 0.946COTD+0.299	Mechanically ventilated patients after liver transplantation
Su(1) 2008[65]	15	USCOM	AoV	-0.51	0.72	0.11	7.2	0.995	COUS = 0.923COTD+0.569	Mechanically ventilated patients after liver transplantation
Su-1 2008[66]	10	USCOM	AoV	-1.06	1.10	0.02	13.0	0.980		living donor liver transplants
Tan 2005[67]	22	USCOM	TPF/TAF	-1.43	1.78	0.18				mechanically ventilated patients following cardiac surgery
Tchorz 2012[13]	29	TTE	AoV			-1.00		0.600		critically ill and/or injured patients admitted to a adult trauma center
Temporelli 2010[68]	43	TTE	LVOT	-0.89	0.78	0.40		0.940	COUS = 1.21COTD+0.016*	advanced heart failure (NYHA Ⅲ-Ⅳ)
Thom 2009[69]	89	USCOM	AoV	-3.01	2.83	-0.10	28.3			ICU patients
Tibbals 1988[70]	18	TTE	AA	-0.33	0.25	0.04		0.970	COUS = 1.03COTD-0.02	Children after cardiac surgery on CPB
Tsutsui 2009[71]	29	UD	AV loop	-1.04	1.08	-0.02	23.5	0.910	COUS = 1.11COTD-0.47	Adult patients undergoing abdominal surgery.
Van den Oever 2007[72]	22	USCOM	AoV	-3.66	2.08	-0.79				ASA-PS4 cardiac surgical patients
Van den Oever(1) 2007[72]	22	USCOM	PA	-3.30	2.97	-0.17				ASA-PS4 cardiac surgical patients
Warth 1984[73]	16	TTE	AoV	-2.01	1.87	-0.07	13.0	0.920	COUS = 0.346COTD+3.33	suspected valvular aortic stenosis
Wong 2008[74]	12	USCOM	TPF/TAF	-1.47	2.25	-0.40		0.896		Liver transplantation.
Wong 1990[75]	58	TTE	AoV	-2.24	0.86	-0.69		0.900	COUS = 0.90COTD+ 0.01	ICU patients and volunteers
Wong-1 1990[76]	56	TTE	AoV	-4.61	3.03	-0.80		0.510	COUS = 0.53COTD+ 2.38	Mechanically ventilated, cardiac surgery, aortic surgery, dysrhythmias or sepsis patients
Zhao 2003[77]	30	TEE	LVOT	-0.79	0.93	-0.09	24.0	0.870		CABG
Zhao(1) 2003[77]	30	TEE	RVOT	-1.10	0.86	-0.18	23.0	0.880		CABG
Zhao(2) 2003[77]	30	TEE	AoV	-0.65	0.99	0.11	27.0	0.840		CABG

*US* ultrasound, *CCA* common carotid artery, *LOA* limits of agreement, *PE* percentage error, *R* linearly dependent coefficient, *PA* pulmonary artery, *TD* thermodilution technique, *COUS* cardiac output measurement by ultrasound, *COTD* cardiac output measurement by thermodilution, *USCOM* ultrasonic cardiac output monitor, *TTE* transthoracic echocardiography, *TEE* transoesophageal echocardiography, *UFP* ultrasonic flow probe, *UD* ultrasound dilution, *RT-3DE* real-time 3D echocardiography, *POCUS* point-of-care ultrasound, *LVOT* left ventricular outflow tract, *RVOT* right ventricular outflow tract, *ACM* automated cardiac output measurement, *AA* ascending aorta, *DA* descending aorta, *AOV* aortic valve, *MV* mitral valve, *TPF* transpulmonary blood flow, *TAF* transaortic blood flow, *AV loop* arteriovenous loop, *cwt* continuous wave Doppler transverse plane, *pwt* pulsed wave Doppler transverse plane, *cw*l continuous wave Doppler longitudinal plane, *pwl* pulsed wave Doppler longitudinal plane, *PiCCO* pulse indicator continuous cardiac output, *CABG* coronary artery bypass surgery, *ASA-PS4* The American Society of Anesthesiologists Physical Status Score 4 class, *CPB* Cardiopulmonary bypass, *CHF* Congestive heart failure, *PH* Pulmonary hypertension, *MVD* Mitral valve disease, *NR* not reported.

*The equation was derived from the transformation.

### Characteristics and qualities of included studies

**Fig 2:**
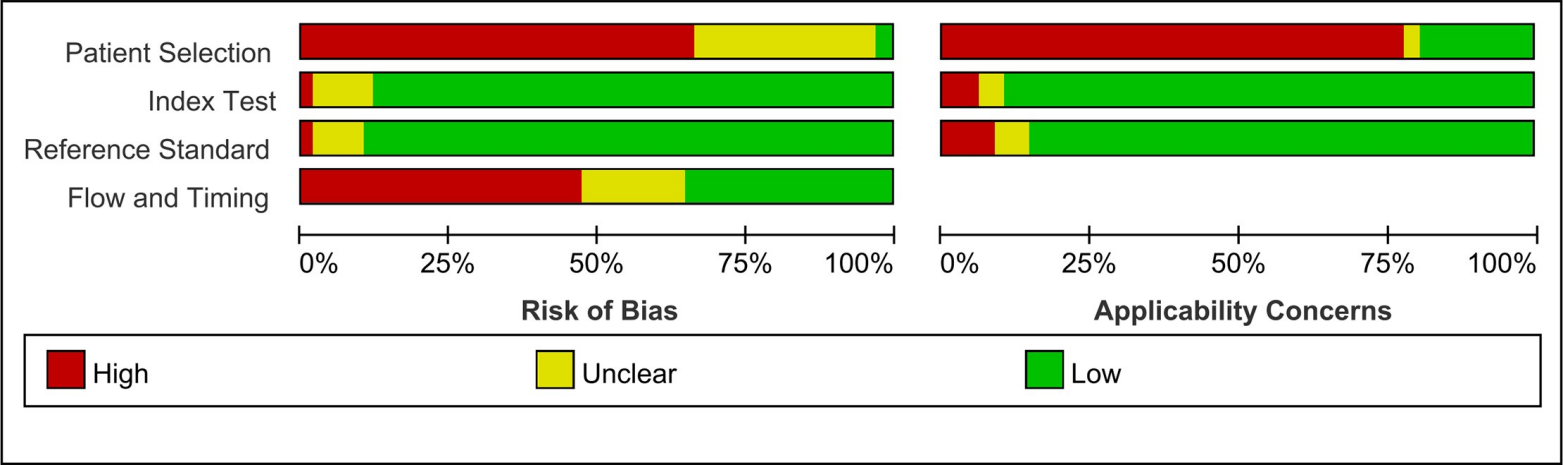
Diagram demonstrating the studies’ percentage compliance, risk of bias, and applicability concerns.

Sixty-nine articles involving 1996 subjects were included. Of these studies, the number of patients ranged from 6 to 89. CO measurements were performed using TTE in 19 studies[2, 13, 23, 26, 27, 41, 44–46, 49–51, 58, 64, 68, 70, 73, 75], TEE in 24 [6–8, 18, 20, 30–32, 34, 42, 47, 48, 52–57, 59–63, 77], USCOM in 14 [4, 19, 22, 25, 28, 39, 40, 43, 65–67, 69, 72, 74], UD in 4 [29, 33, 36, 71], and other types of echocardiography in 7 [10, 21, 24, 35, 37, 38, 61]. CO measurements were performed in the AA in 13 studies [2, 4, 19, 24, 27, 28, 32, 35, 37, 41, 45, 47, 70], AOV in 18 [6, 13, 22, 23, 30, 43, 44, 46, 48, 51, 55, 65, 66, 69, 72, 73, 75–77], and LVOT in 12 [8, 18, 21, 31, 34, 49, 50, 54, 57, 64, 68, 77] with VTI. Further, CO measurements were performed in 5 studies in the PA [2, 5, 53, 60, 61], and CO measurement using the Simpson method in 3[20, 38, 56]. Of these studies, Bland-Altman analyses were used in 56 studies, and the LOA and bias were available in 59. Linear regression analyses were used in 54 studies, and 35 regression equations were acquired. Correlation analyses were used in most studies, and the correlation coefficient (R value) was obtained except for the other 15 studies (Table 2). The methodological qualities of the included studies were evaluated according to the tailored QUADAS-2. The results are shown in Fig 2 and S1 Fig.

### CO evaluation using different types of Doppler

Of these included studies, there were 41 studies with 49 CO measured results, and 1522 patients were included in the meta-analysis; no significant differences were observed between US and TD (random effects model: MD, -0.14; 95% confidence interval [CI], -0.30 to 0.02; *P* = 0.08). The subgroup analyses were conducted using different types of echocardiography techniques. In 19 of the TTE studies, 12 with 14 sets of data and 290 patients were included in the meta-analysis. The result showed no significant differences between TTE and TD (random effects model: MD, -0.28; 95% CI, -0.71 to 0.15; *P* = 0.20). In 24 of the TEE studies, 13 with 19 sets of data and 606 patients were included in the meta-analysis. The result showed that no significant differences were observed between TEE and TD (random effects model: MD, 0.00; 95% CI, -0.12 to 0.11; *P* = 0.98). In 13 of the USCOM studies, 10 with 10 sets of data and 356 patients were included in the meta-analysis. No significant differences were observed between USCOM and TD (random effects model: MD, -0.16; 95% CI, -0.61 to 0.28; *P* = 0.47). No significant differences were observed in four studies between UD and TD (random effects model: MD, 0.00; 95% CI, -0.43 to 0.44; *P* = 0.99), and no significant differences were observed in the other 4 studies between other types of methods and TD (random effects model: MD, -0.56; 95% CI, -1.25 to 0.14; *P* = 0.12) (Table 2 and S2 Fig). The sensitivity analysis showed that no change occurred in the overall effect and subgroup analysis effect when some studies were omitted up to the acceptable heterogeneity (Table 3 and S3 Fig).

**Table 2:** Meta-analyses of the cardiac output measurement by echocardiography (US) vs. thermodilution (TD).

Outcome or Subgroup	Studies	Participants	Heterogeneity		Meta-analysis model	Effect Estimate	
			I^2^	*P*		MD (95%CI)	*P*
1 All	43	1522	67	<0.01	IV, Random	-0.14 [-0.30, 0.02]	0.08
1.1 TTE	12	290	85	<0.01	IV, Random	-0.28 [-0.71, 0.15]	0.20
1.2 TEE	13	606	0	0.98	IV, Random	0.00 [-0.12, 0.11]	0.98
1.3 USCOM	10	356	71	0.001	IV, Random	-0.16 [-0.61, 0.28]	0.47
1.4 UD	4	113	0	1.00	IV, Random	0.00 [-0.43, 0.44]	0.99
1.5 Others types	4	157	73	0.01	IV, Random	-0.56 [-1.25, 0.14]	0.12
2 All	43	1446	68	<0.01	IV, Random	-0.15 [-0.31, 0.00]	0.06
2.1AA	6	202	64	0.01	IV, Random	-0.37 [-0.71, -0.01]	0.05
2.2 AOV	15	463	75	<0.01	IV, Random	-0.03 [-0.31, 0.25]	0.83
2.3 LVOT	8	418	55	0.01	IV, Random	-0.06 [-0.32, 0.21]	0.67
2.4 PA	2	44	0	0.97	IV, Random	-0.09 [-0.63, 0.44]	0.73
2.5 AV loop	3	87	0	1.00	IV, Random	-0.01 [-0.46, 0.45]	0.97
2.6 TPF/TAF	3	102	0	0.81	IV, Random	0.05 [-0.58, 0.68]	0.88
2.7 Others sites	6	130	77	<0.01	IV, Random	-0.53 [-1.40, 0.33]	0.23

*TTE* transthoracic echocardiography, *TEE* transoesophageal echocardiography, *USCOM* ultrasonic cardiac output monitor, *UD* ultrasound dilution, *AOV* aortic valve, *LVOT* left ventricular outflow tract, *AA* ascending aorta, *PA* pulmonary artery, *AV loop* arteriovenous loop, *TPF* transpulmonary blood flow, *TAF* transaortic blood flow, *IV* inverse variance, *MD* mean difference, *CI* confidence interval

**Table 3:** Sensitivity analysis of high heterogeneity outcomes in meta-analysis.

Heterogeneity outcomes	Participants	Omitted studies	Heterogeneity		Meta-analysis model	Outcomes	
			I^2^	P		MD (95%CI)	*P*
3 Types of Doppler	1407		0%	0.99	IV, Fixed	0.00 [-0.08, 0.09]	0.94
3.1 TTE	228		30%	0.28	IV, Fixed	0.14 [-0.12, 0.41]	0.28
3.2 TEE	606		0%	1.00	IV, Fixed	0.00 [-0.12, 0.11]	0.98
3.3 USCOM	336		0%	0.94	IV, Fixed	-0.03 [-0.18, 0.25]	0.75
3.4 UD	113		0%	1.00	IV, Fixed	0.00 [-0.43, 0.44]	0.99
3.5 Others	124		0%	0.98	IV, Fixed	-0.19 [-0.47, 0.09]	0.18
4 Sites	1351		0%	0.77	IV, Fixed	-0.02 [-0.11, 0.06]	0.63
4.1 AA	192		15%	0.32	IV, Fixed	-0.20 [-0.39, -0.00]	0.04
4.2 AOV	431		0%	0.65	IV, Fixed	0.02 [-0.15, 0.19]	0.80
4.3 LVOT	385		0%	0.57	IV, Fixed	0.06 [-0.08, 0.19]	0.41
4.4 PA	44		0	0.97	IV, Fixed	-0.09 [-0.63, 0.44]	0.73
4.5 AV loop	87		0	1.00	IV, Fixed	-0.01 [-0.46, 0.45]	0.97
4.6 TPF/TAF	102		0	0.81	IV, Fixed	0.05 [-0.58, 0.68]	0.88
4.7 Others	110		0%	0.98	IV, Fixed	-0.15 [-0.50, 0.20]	0.40

*TTE* transthoracic echocardiography, *TEE* transoesophageal echocardiography, *USCOM* ultrasonic cardiac output monitor, *UD* ultrasound dilution, *AOV* aortic valve, *LVOT* left ventricular outflow tract, *AA* ascending aorta, *PA* pulmonary artery, *AV loop* arteriovenous loop, *TPF* transpulmonary blood flow, *TAF* transaortic blood flow, *IV* inverse variance, *MD* mean difference, *CI* confidence interval

### CO evaluation at different sites

In six studies, the AA was used to measure CO by Doppler, and six studies were included in the meta-analysis. Significant differences were observed in the use of US at AA with TD (random effects model: MD, -0.37; 95% CI, -0.71 to -0.11; *P* = 0.05). Moreover, no significant differences were observed in the use of US at AOA (random effects model: MD, -0.03; 95% CI, -0.31 to 0.25; *P* = 0.83), LVOT (random effects model: MD, -0.06; 95% CI, -0.32 to 0.21; *P* = 0.67), PA (random effects model: MD, -0.09; 95% CI, -0.63 to 0.44; *P* = 0.73), AV loop (random effects model: MD, -0.01; 95% CI, -0.46 to 0.45; P = 0.97), TPF/TAF (random effects model: MD, 0.05; 95% CI, -0.58 to 0.68; *P* = 0.88), and other sites (random effects model: MD, -0.53; 95% CI, -1.40 to 0.33; *P* = 0.23) (Table 2 and S4 Fig). The sensitivity analysis showed no changes in the overall effect and the subgroup analyses (Table 3 and S5 Fig).

### Bland-Altman analyses and regression analyses

In all studies, the median of bias between US and TD was -0.12 (ranged from -2.50 to 3.04 L/min). The median of LOA was 0.94 L/min (ranged from ±0.05 to ±4.72 L/min). Twenty-eight studies reported that the PE and the median were 24.3% (ranged from 7.2% to 53%). The median of R (correlation coefficient) was 0.827 (ranged from 0.140 to 0.998). The slope ranged from 0.126 to 1.58, and the intercept ranged from -1.91 to 3.55 in the 35 regression equations (Table 1).

### Publication bias

The funnel plot was roughly symmetrical (S5 Fig). Egger’s test revealed no publication bias in the literature (*P* = 0.500) (S6 Fig).

## Discussion

In this systematic review, we included 68 studies, of which 43 studies reported data on CO measurement and were included in the meta-analysis. The overall effect showed that no significant difference was observed between echocardiography and TD in measuring CO; the subgroup analysis showed no significant differences in the different types. In all sites, the difference was founded only in AA. Further, the sensitivity analysis showed no change in the results. However, there was a wide range in bias, LOA, PE, and correlation coefficient of the two technologies and was beyond the clinically acceptable range in some studies.

In these different types of echocardiography, the sensitivity analysis showed that the TEE, USCOM, and UD had hairline bias (≤0.1 L/min) with TD; TEE had the lowest standard error and maximum weight followed by TTD. The UD had a small mean difference, but with wide 95% CI. CO could be easily overestimated using TTE instead of using TD, and underestimated using other types of echocardiography (ACM, NICO, point-of-care US, and Doppler PA catheter) despite the differences not having statistical significance. Therefore, we speculate that TEE was the preferred method because it can obtain more accurate results in the measurement of CO. Considering that TEE is semi-invasive and UD can only be used for specific population, USCOM can be the first choice for noninvasive echocardiography for the measurement of CO.

The sites of the ultrasonic probe will also have an effect on the test results. In our meta-analysis, no statistical difference was found except for the measurement of CO in the AA. The lowest mean difference of CO comes from PA, compared with the TD, followed by AV loop. However, the method of measuring PA CO used a PA catheter ultrasound probe and was an invasive procedure [59]. The AV loop used in UD is also based on an invasive procedure[28, 33, 35, 68]. In these studies, more researchers were willing to measure CO in the AOV and LVOT, with mean differences of <0.1 L/min and a narrower LOA. Therefore, the AOV and LVOT as the recommended sampling locations for CO detection are feasible. This finding is also consistent with the recommendation of the American ultrasound guidelines [78].

In all these studies, the largest bias and LOA (bias = 3.04 L/min, LOA = ±9.44 L/min) were found in a study with the ABCOM 1 transtracheal Doppler (TTD) versus TD [34] and with the lowest correlation coefficient (R = 0.14). TTD system requires a special TTD endotracheal tube, in which the tip was embedded with an ultrasonic probe; it can only be used in patients with mechanical ventilation. In this study, seven patients with 36 simultaneous measurements were compared. We speculated that the TTD measurements accounted for most of the between-technique variability. Obtaining and maintaining good Doppler signals were difficult and time-consuming in TTD and were considered possible causes of error. Hausen et al. [37] also compared TTD and TD. They suggested that the TTD system does not provide accurate CO determinations (bias = 1.70 L/min, LOA = ±3.29 L/min, R = 0.248) and that several reasons can affect its accuracy and restrict its wider use, such as cuff deflation for >10 min, which cannot be tolerated by ICU doctors, sensitivity to patient movement, and that an optimal signal cannot often be attained if the probe is not in the appropriate place.

The bias in three studies[2, 21, 25] was > 2.0 L/min. One of these studies[21] used the ACM to monitor the CO in patients with high cardiac output (pregnant and pre-eclamptic women) and found that it was inaccurate compared with TD. Another study[2] found that the mean difference was larger in the spontaneous ventilation state than the intermittent positive pressure ventilation and apnea state. One possible reason was that the patients were not sedated during spontaneous ventilation; thus, the CO was increased. Cariou et al. [25] compared the descending aortic blood flow using a pulse Doppler velocimeter with CO. Although the authors thought that the descending aortic blood flow determination had good correlation and consistency with TD in CO and that descending aortic blood flow provided a reliable noninvasive tool for estimating CO, the mean difference was obviously due to the descending aortic blood flow as a fraction of the CO.

Critchley and Critchley [79] thought that it can be acceptable clinically when the PE is <30%. They suggest that if the PE between the two methods is ≤±30%, then the two methods are interchangeable. In our studies, although the median of PE was 23.8%, the PE of the six studies [2, 8, 18, 24, 43, 51] was >30%. Missant et al. [51] used Doppler echocardiography during off-pump coronary artery bypass grafting and believed that Doppler echocardiography was not always feasible when the heart was displaced from the esophagus and had lower accuracy; The accuracy in CO measurement may have been affected in three studies that included special patients or scenarios (Knirsch et al. [43] had used USCOM in children with congenital heart disease; Botero et al. [24] used NICO during cardiopulmonary bypass; and Castor et al. [2] used NICO in patients with low sedation levels during spontaneous ventilation). Moller-Sorensen et al. [18] thought that the possible explanation was that the SV is calculated from two variables (LVOT, CSA, and the VTi); measurements were made irrespective of the ventilatory cycle, arrhythmias, and the patients with different scenarios. Therefore, we should pay more attention to the evaluation of ultrasound CO results, when the cardiac function or physiological structural change occurs in some patients with heart disease or in special situations.

Moreover, imprecision in echocardiography CO measurements may be induced by technical or operator factors. By improving the operation level, repeated measurement may reduce the measurement error. In our meta-analysis, self-control was used in all studies, and most of the studies used repeated measurements and blinding methods. Therefore, the quality of literature was not evaluated.

Other limitations of this study include the following: (1) no further subgroup analysis was conducted on the research subjects and disease types due to the limitation of data integrity and the diversity of diseases; (2) determination of the best CO test method was difficult, as both have advantages and limitations; (3) the linear equations were not overfitted because finding a general linear equation to express the relationship between US and TD in CO measurement for the inconsistency of the research subjects, ultrasonic type, and sites is difficult.

## Conclusions

This systematic review and meta-analysis showed that the overall effect of the CO measurements by echocardiography or TD has no significant difference. TEE can be the preferred method with accurate results and USCOM can be a good choice for its noninvasiveness in CO measurement; the AOV and LVOT can be the recommended sampling location. However, in some special scenarios, such as high CO, low sedation, or with physiological structural changes, the accuracy of CO measurement by echocardiography is questionable.

## Supporting information

S1 Table Tailored QUADAS-2 to assess risk of bias and applicability judgments. (DOC)

S1 Checklist PRISMA 2009 Checklist. (DOC)

S1 Fig Diagram demonstrating the assessment of bias for each study included in our analysis. (TIF)

S2 Fig Forest plot of comparison in cardiac output measurement and subgroup analysis with different types of echocardiography (US) vs. thermodilution (TD). *IV* inverse variance, *CI* confidence interval, *MD* mean difference.
(TIF)

S3 Fig Forest plot of sensitivity analysis of cardiac output measurement and subgroup analysis with different types of echocardiography (US) vs. thermodilution (TD). *IV* inverse variance, *CI* confidence interval, *MD* mean difference.
(TIF)

S4 Fig Forest plot of comparison of cardiac output measurement and subgroup analysis with different sites for echocardiography (US) vs. thermodilution (TD). *IV* inverse variance, *CI* confidence interval, *MD* mean difference.
(TIF)

S5 Fig Forest plot of sensitivity analysis of cardiac output measurement and subgroup analysis with different sites for echocardiography (US) vs. thermodilution (TD). *IV* inverse variance, *CI* confidence interval, *MD* mean difference.
(TIF)

S6 Fig Funnel plot for publication bias in cardiac output measurement between echocardiography (US) vs. thermodilution (TD) (Egger’s test, *P* = 0.500). (TIF)
