# Supplementary material for: Cardiac output measurements via echocardiography versus thermodilution: A systematic review and meta-analysis
Source: PLoS One. 2019 Oct 3;14(10):e0222105. doi: 10.1371/journal.pone.0222105 (PMC6776392; doi:10.1371/journal.pone.0222105)
Supplement: S1 Table — (DOC) [file pone.0222105.s001.doc]

Table S1. A tailored QUADAS-2 for Risk of Bias and Applicability Judgments.

| DOMAIN 1: PATIENT SELECTION |  |
| --- | --- |
| A. Risk of Bias |  |
| 1. Was a consecutive or random sample of patients enrolled? | Yes/No/Unclear |
| 2. Did the study avoid inappropriate exclusions? | Yes/No/Unclear |
| 3. Was a self-control study? | Yes/No/Unclear |
| Could the selection of patients have introduced bias? | RISK: LOW/HIGH/UNCLEAR |
| B. Concerns regarding applicability |  |
| Is there concern that the included patients do not match the review question? | CONCERN: LOW/HIGH/UNCLEAR |
| DOMAIN 2: INDEX TEST(S) |  |
| A. Risk of Bias |  |
| 1. Were the index test results interpreted without knowledge of the results of the reference standard? | Yes/No/Unclear |
| 2. Was the execution of the index test described in sufficient detail to permit replication of the test? | Yes/No/Unclear |
| Could the conduct or interpretation of the index test have introduced bias? | RISK: LOW/HIGH/UNCLEAR |
| B. Concerns regarding applicability |  |
| Are there concerns that the index test, its conduct, or interpretation differ from the review question? | CONCERN: LOW/HIGH/UNCLEAR |
| DOMAIN 3: REFERENCE STANDARD |  |
| A. Risk of Bias |  |
| 1. Were the reference standard results interpreted without knowledge of the results of the index tests? | Yes/No/Unclear |
| 2. Was the reference standard independent of the index test (i.e. the index test did not form part of the reference standard)? | Yes/No/Unclear |
| 3. Was the execution of the reference standard described in sufficient detail to permit its replication? | Yes/No/Unclear |
| Could the reference standard, its conduct, or its interpretation have introduced bias? | RISK: LOW/HIGH/UNCLEAR |
| B. Concerns regarding applicability |  |
| Are there concerns that the target condition as defined by the reference standard does not match the question? | CONCERN: LOW/HIGH/UNCLEAR |
| DOMAIN 4: FLOW AND TIMING |  |
| A. Risk of Bias |  |
| 1. Was there an appropriate interval between index test and reference standard? | Yes/No/Unclear |
| 2. Did all patients receive the same reference standard? | Yes/No/Unclear |
| 3. Were all patients included in the analysis? | Yes/No/Unclear |
| Could the patient flow have introduced bias? | RISK: LOW/HIGH/UNCLEAR |
