# Supplementary figures and images for: Cardiac output measurements via echocardiography versus thermodilution: A systematic review and meta-analysis
Source: PLoS One. 2019 Oct 3;14(10):e0222105. doi: 10.1371/journal.pone.0222105 (PMC6776392; doi:10.1371/journal.pone.0222105)

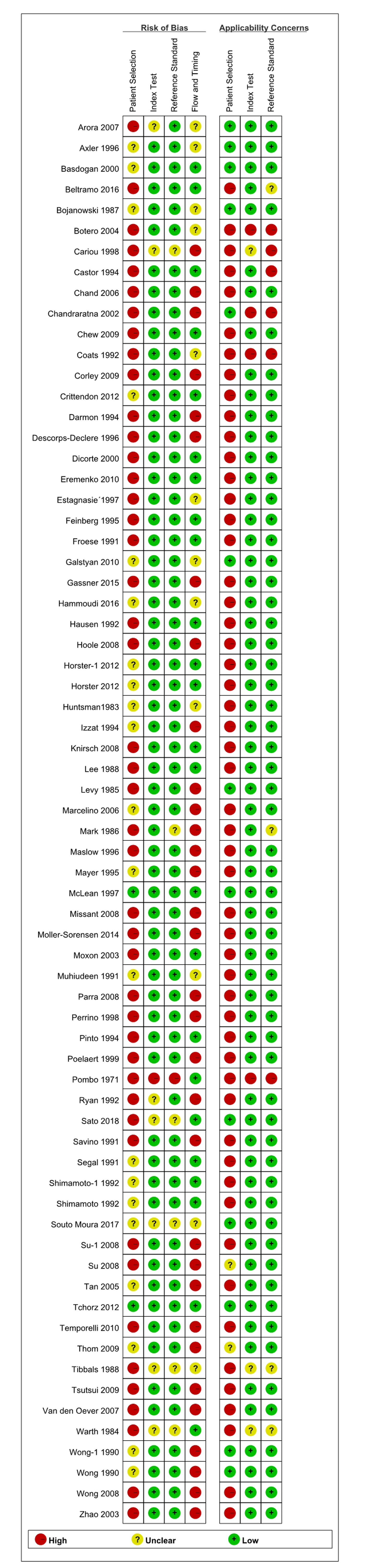

Supplement: S1 Fig — (TIF) [file pone.0222105.s003.tif]

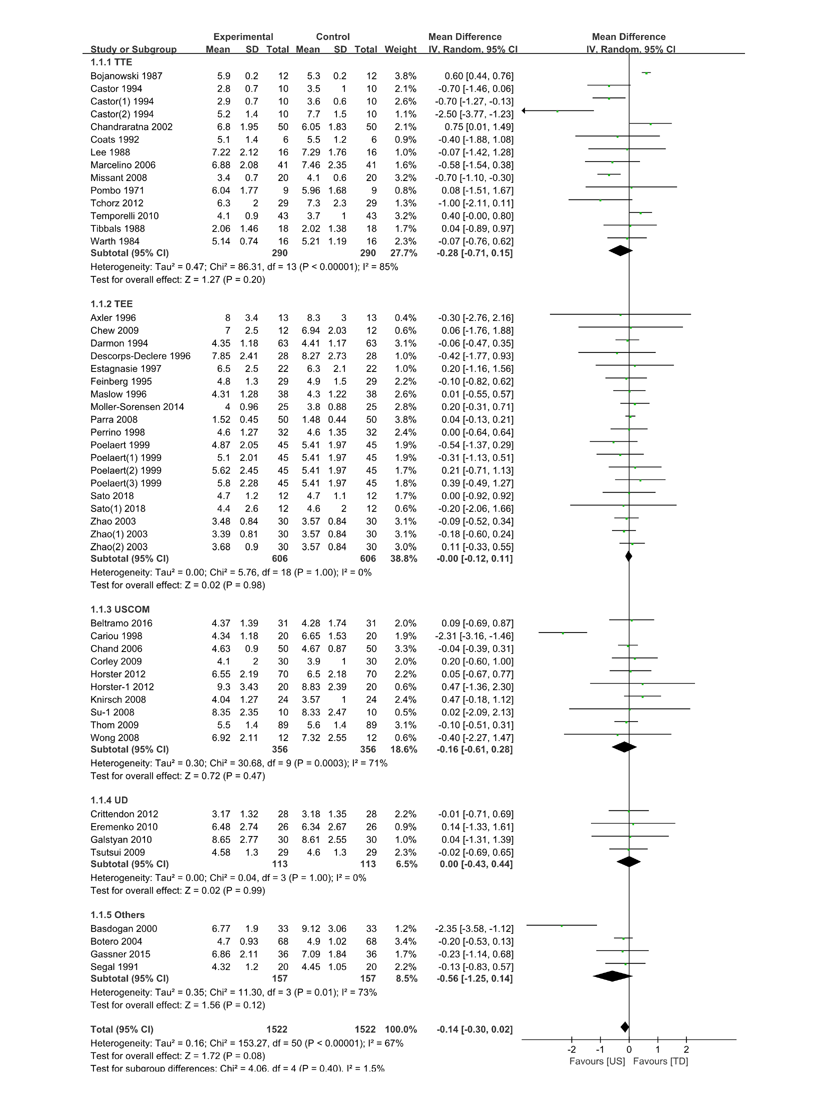

Supplement: S2 Fig — IV inverse variance, CI confidence interval, MD mean difference. (TIF) [file pone.0222105.s004.tif]

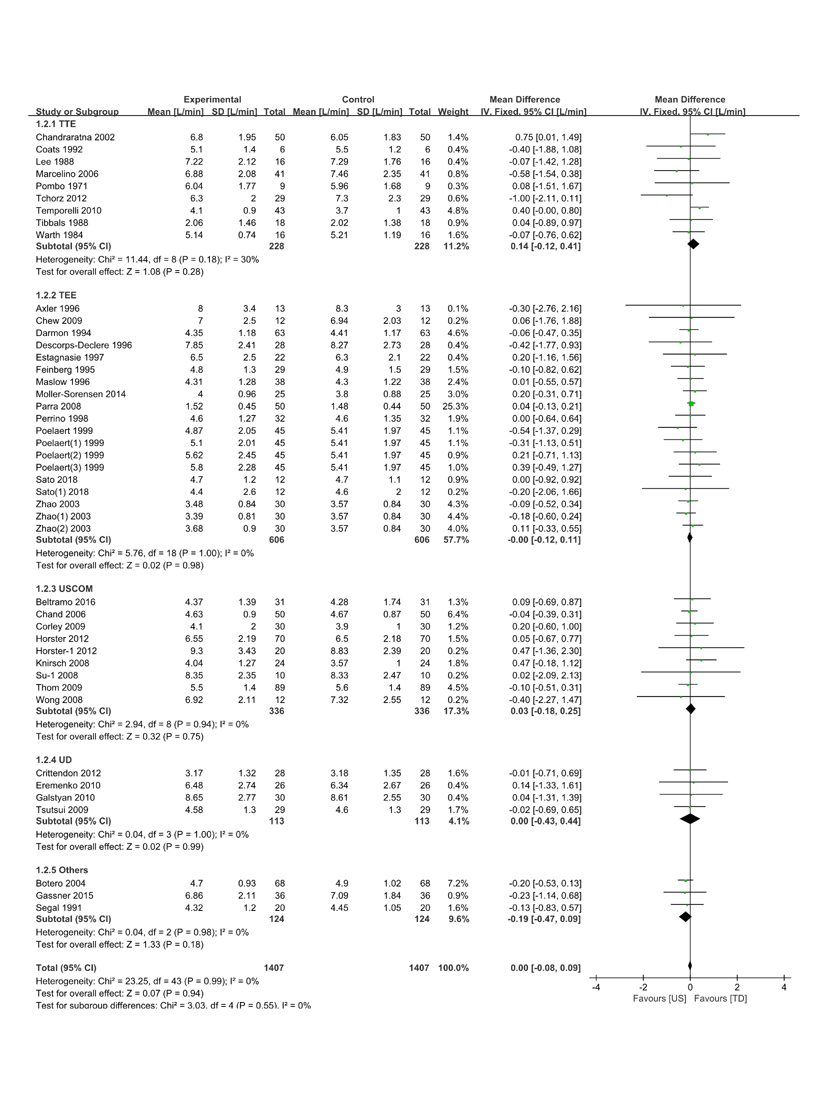

Supplement: S3 Fig — IV inverse variance, CI confidence interval, MD mean difference. (TIF) [file pone.0222105.s005.tif]

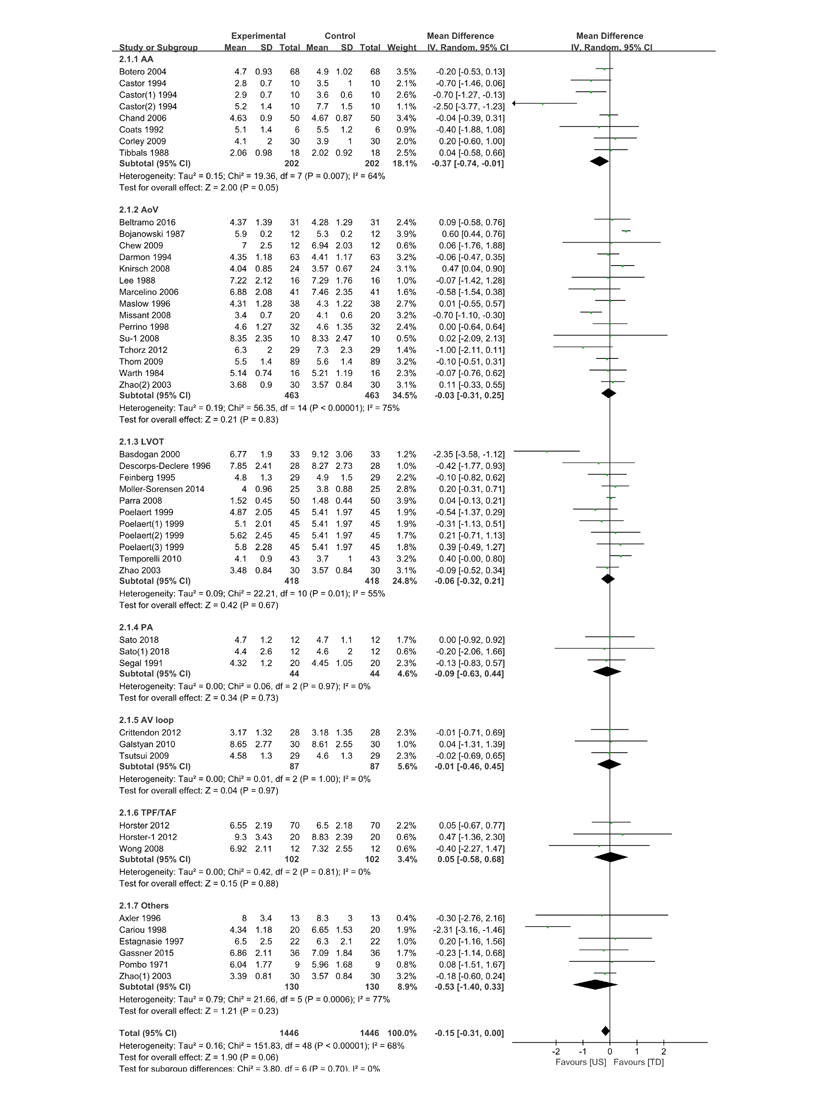

Supplement: S4 Fig — IV inverse variance, CI confidence interval, MD mean difference. (TIF) [file pone.0222105.s006.tif]

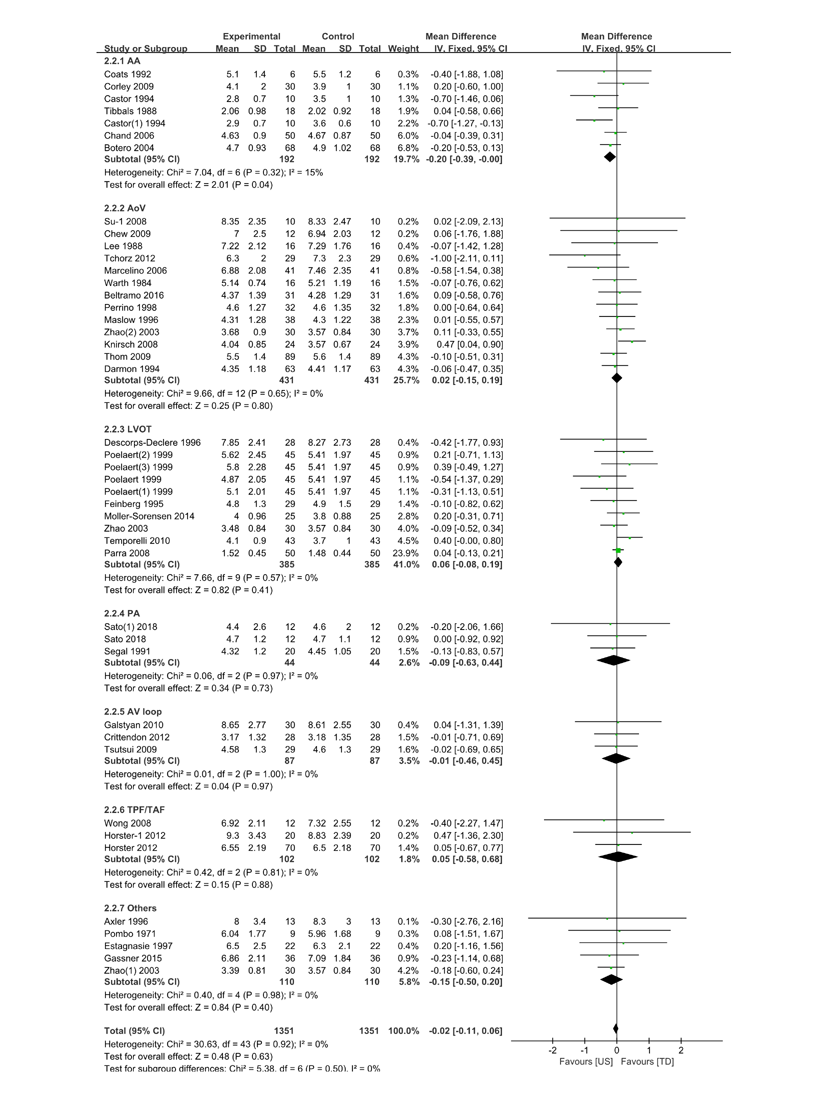

Supplement: S5 Fig — IV inverse variance, CI confidence interval, MD mean difference. (TIF) [file pone.0222105.s007.tif]

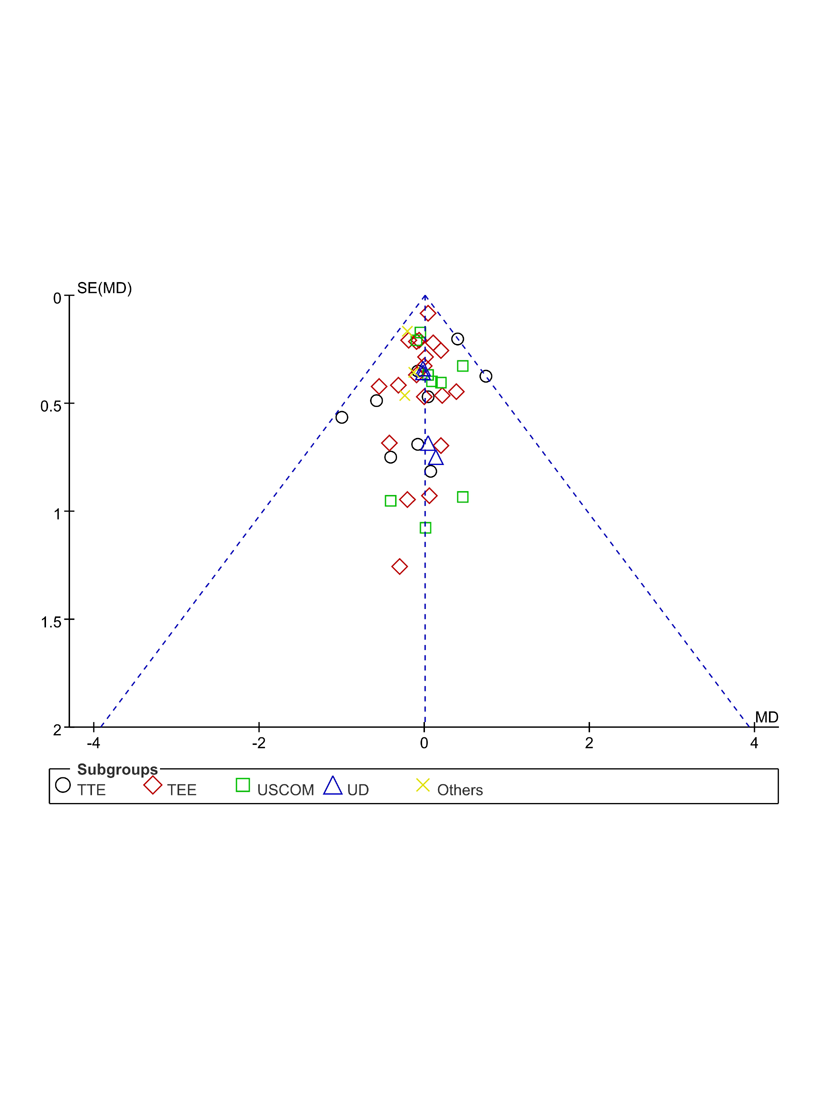

Supplement: S6 Fig — (TIF) [file pone.0222105.s008.tif]
